# Dopamine synthesis and transport: current and novel therapeutics for parkinsonisms

**DOI:** 10.1042/BST20231061

**Published:** 2024-05-30

**Authors:** Mary Dayne Sia Tai, Gloria Gamiz-Arco, Aurora Martinez

**Affiliations:** 1 Department of Biomedicine, University of Bergen, 5009 Bergen, Norway; 2 K.G. Jebsen Center for Translational Research in Parkinson's Disease, University of Bergen, 5020 Bergen, Norway; 3 Neuro-SysMed, Department of Neurology, Haukeland University Hospital, 5021 Bergen, Norway

**Keywords:** dopamine deficiencies, inherited disorders, Parkinson's disease, parkinsonism, small molecules, therapy

## Abstract

Parkinsonism is the primary type of movement disorder in adults, encompassing a set of clinical symptoms, including rigidity, tremors, dystonia, bradykinesia, and postural instability. These symptoms are primarily caused by a deficiency in dopamine (DA), an essential neurotransmitter in the brain. Currently, the DA precursor levodopa (synthetic L-DOPA) is the standard medication to treat DA deficiency, but it only addresses symptoms rather than provides a cure. In this review, we provide an overview of disorders associated with DA dysregulation and deficiency, particularly Parkinson's disease and rare inherited disorders leading predominantly to dystonia and/or parkinsonism, even in childhood. Although levodopa is relatively effective for the management of motor dysfunctions, it is less effective for severe forms of parkinsonism and is also associated with side effects and a loss of efficacy over time. We present ongoing efforts to reinforce the effect of levodopa and to develop innovative therapies that target the underlying pathogenic mechanisms affecting DA synthesis and transport, increasing neurotransmission through disease-modifying approaches, such as cell-based therapies, nucleic acid- and protein-based biologics, and small molecules.

## Introduction

Parkinsonism refers to a constellation of clinical symptoms and manifestations primarily associated to motor dysfunction, such as rigidity, tremors, dystonia, bradykinesia and postural instability [1], and is considered as the predominant form of movement disorder in adults. The development of parkinsonism can be related to many factors such as traumatic brain injuries [2], inherited disorders [3], drugs [4] or prolonged exposure to toxins [5], but most cases are associated with Parkinson's disease (PD). In a molecular context, parkinsonism can result from oxidative stress or dysfunctions in the mitochondria, ubiquitin-proteasome system, endosomal trafficking, lysosomal autophagy or monoamine neurotransmission [3]. Regardless of the underlying molecular cause, patients with parkinsonism typically have a deficiency in dopamine (DA), due to direct effects from gene variants leading to deficiency in the function and/or levels of proteins involved in DA production, metabolism or signaling, or due to the indirect impacts of other dysfunctional cellular pathways that lead to the death of dopaminergic neurons, as occurs in PD.

**Figure 1.**
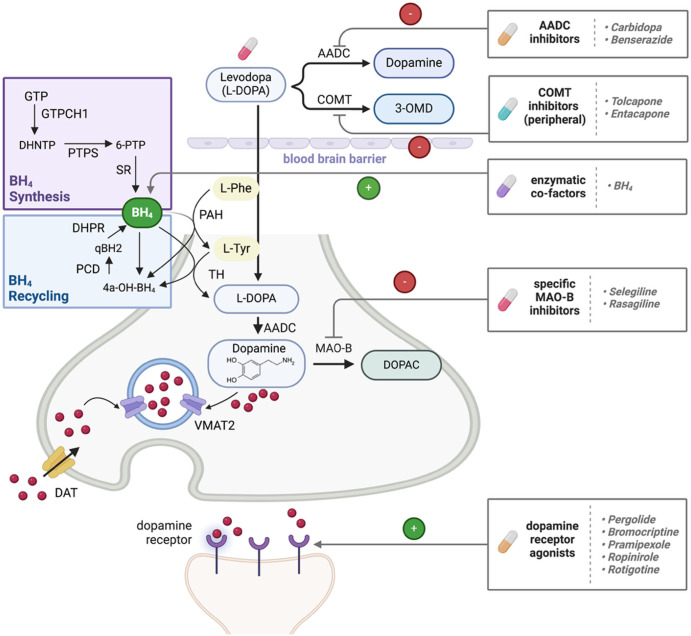
Simplified overview of the pathways involved in the biosynthesis, metabolism and transport of DA, highlighting the targets of current therapeutic approaches designed to enhance DA signaling. DA is synthesized by PAH, TH and AADC, and is then packed into synaptic vesicles by VMAT2 and released into the synapse for binding to receptors on the postsynaptic neuron. Residual DA in the synaptic cleft undergoes reuptake by DAT and is repackaged in synaptic vesicles or metabolized by monoamine oxidase (MAO) to 3,4-dihydroxyphenylacetic acid (DOPAC), which can be exported to astrocytes and microglia for further metabolism (see Meiser et al. [18] and Winner et al. [17]). See also Meiser et al. [18] for further reading on an alternative DA biosynthesis pathway. Current treatments for DA deficiencies that boost DA signaling involve administration of levodopa, inhibitors of AADC, COMT and/or MAO-B, BH_4_ (in BH_4_-deficiencies) and DA agonists. Abbreviations: 3-OMD, 3-O-methyldopa; 4a-OH-BH_4_, pterin-4-alpha-carbinolamine; 6-PTP, 6-pyruvoyl tetrahydropterin; AADC, aromatic acid decarboxylase; BH_4_, (6R)-L-erythro-5,6,7,8-tetrahydrobiopterin; COMT, catechol-O-methyltransferase; DAT, dopamine transporter; DHNTP, 7,8-dihydroneopterin triphosphate; DHPR, dihydropteridine reductase; DOPAC, 3,4-dihydroxyphenylacetic acid; GTPCH1, GTP cyclohydrolase; GTP, guanosine triphosphate; L-DOPA, L-3,4-dihydroxyphenylalanine; MAO-B, monoamine oxidase B; PAH, phenylalanine hydroxylase; PCD, pterin-4-alpha-carbinolamine dehydratase; PTPS, 6-pyruvoyl tetrahydropterin synthase; qBH2, dihydrobiopterin quinoid; SR, sepiapterin reductase; TH, tyrosine hydroxylase; VMAT2, vesicular monoamine transporter 2.

DA is a monoamine neurotransmitter and hormone that is essential for signaling in the central and peripheral nervous system [6]. DA is responsible for memory, cognition, reward, learning and movement and thus, low DA levels contribute to parkinsonism. Due to the importance of DA for proper nervous system function, its synthesis, trafficking and metabolism must be tightly controlled. Monoamine neurotransmitters such as DA are synthesized from the amino acids L-Phe, L-Tyr and L-Trp in pathways involving the aromatic amino acid hydroxylases (AAAHs), a family of specialized enzymes comprised of phenylalanine hydroxylase (PAH), tyrosine hydroxylase (TH) and tryptophan hydroxylases 1 and 2 (TPH1 and TPH2), that require a catalytic non-heme ferrous iron and tetrahydrobiopterin (BH_4_) as cofactor [7]. The TPHs catalyze the rate-limiting step in serotonin biosynthesis and PAH hydroxylates L-Phe to L-Tyr, mainly in liver. In dopaminergic neurons, TH catalyzes the hydroxylation of L-Tyr to L-3,4-dihydroxyphenylalanine (L-DOPA), which is converted to DA by aromatic acid decarboxylase (AADC) (Figure 1).

The conversion of L-Tyr to L-DOPA is the rate-limiting step in DA synthesis [8]. Due to the cellular toxicity associated with excess DA, TH activity is tightly-regulated at both the transcriptional (e.g. regulation of mRNA levels, alternative splicing and modulation of mRNA stability) [9] and post-transcriptional levels (e.g. TH phosphorylation and dephosphorylation [10], protein-protein interactions, with GTPCH1, 14-3-3, and DNAJC12, among others [11–14] and DA feedback inhibition in interplay with phosphorylation at Ser40 [15,16]). Surplus of DA in the synapse is also taken back into the presynaptic terminal by the DA transporter (DAT) and is subsequently either metabolized by enzymes such as monoamine oxidase (MAO) or reloaded into vesicles by the monoamine transporter 2 (VMAT2) (Figure 1). Astrocytes and microglia have also been shown to be involved in further DA metabolism [17,18], and thus serve as disease modifiers in PD [19]. Furthermore, alternative pathways of DA synthesis from the precursor 3-methoxytyramine that result in the modulation of DA levels *in vivo* have been described for gut microbiota [20].

In the case of PD, the loss of DA-producing neurons is caused by a complex interplay between genetic and environmental factors with mechanisms that are still unclear. Variants in several genes have been associated with PD (*GBA*, *LRRK2*, *PARK7*, *PINK1*, *PRKN*, *SNCA*), but only ∼10% of reported cases have genetic etiology [21,22]. In the case of genetic errors in proteins directly involved in DA signaling, variants often lead to instability and misfolding, loss of function (loss of activity and/or interactions) and premature degradation, or to gain of function and aggregation, resulting to DA dysregulation and deficiency [23–25]. A list of rare deficiencies leading to decreased levels of DA and/or DA dysregulation that may underlie dystonia and parkinsonism since childhood is outlined in Table 1. Here, we focus on (i) defects in proteins involved in DA biosynthesis or transport; (ii) variants in enzymes involved in the synthesis and regeneration of the AAAH cofactor (BH_4_), and (iii) variants in molecular cochaperones that are involved in maintaining cellular homeostasis of the AAAHs, in particular the Hsp40 cochaperone DNAJC12. Except for the autosomal-dominant form of GTPCH1 (AD-GTPCH1) deficiency, all other deficiencies included in Table 1 are associated with biallelic variants, inherited in a recessive manner. For additional defects related to DA catabolism we refer to Brennenstuhl et al. [26] and Opladen and Hoffmann [27], and for a recent review on mouse models for inherited monoamine neurotransmitter disorders to Thöny et al. [28].

**Table 1.** Overview of main proteins involved in DA synthesis and transport, and associated with inherited disorders that may cause DA deficiencies and underlie parkinsonism

Protein; gene	Function	Pathophysiology
Phenylalanine hydroxylase (PAH); *PAH*	PAH converts L-Phe to L-Tyr (Figure 1). High levels of L-Phe inhibit DA synthesis in the brain by inhibiting L-Tyr transport and TH activity [103,104].	PAH deficiency is associated with phenylketonuria (PKU), characterized by elevated L-Phe levels that can result in neurotoxicity and decreased DA levels. Untreated PKU patients present with intellectual disability, eczematous rash, autism, seizures, and motor deficits [80], although clear parkinsonism is rare in PKU [105]. Worldwide, PKU affects ∼0.45 million individuals [106].
Tyrosine hydroxylase (TH); *TH*	TH catalyzes the conversion of L-Tyr to L-DOPA (Figure 1), the rate-limiting step in DA biosynthesis [107].	TH deficiency (THD) is a rare genetic disorder where patients present a phenotype ranging from L-DOPA responsive dystonia (DRD) to severe encephalopathy. Approximately 100 cases are reported so far [108].
GTP cyclohydrolase 1 (GTPCH1); *GCH1*	GTPCH1 is the first and rate-limiting enzyme in the *de novo* synthesis of BH_4_, responsible for the hydrolysis of GTP to DHNTP (Figure 1).	Symptoms of GTPCH1 deficiency include hypertonia, DRD, gait difficulties, and hyperreflexia [109]. There are both autosomal dominant (AD) and autosomal recessive forms of GTPCH1 deficiency, the former being the most common cause of DRD.
6-pyruvoyl tetrahydropterin synthase (PTPS); *PTS*	PTPS catalyzes the second step in the synthesis of BH_4_, the conversion of DHNTP to 6-PTP (Figure 1).	Individuals with PTPS deficiency present with impaired neurophysiological development, truncal hypotonia, dystonia, bradykinesia, swallowing difficulties and hyperthermia. This is the most common cause of BH_4_ deficiencies (54%) with ∼735 cases reported [110].
Sepiapterin reductase (SR or SPR); *SPR*	SR catalyzes the final two-step reduction in 6-PTP to BH_4_ (Figure 1).	SR deficiency (SRD) is characterized by motor and speech delay, axial hypotonia, dystonia and weakness [111].
Pterin-4-alpha-carbinolamine dehydratase (PCD); *PCBD1*	PCD acts on the regeneration of BH_4_, where it catalyzes the dehydration of the 4a-OH-BH_4_ to qBH_2_ (Figure 1).	PCD deficiency is not associated with significant changes in L-Phe and neurotransmitter levels or severe clinical abnormalities, except for temporary fluctuations in tone [110].
Dihydropteridine reductase (DHPR); *QDPR*	DHPR catalyzes the reduction in qBH_2_ to BH_4_ (Figure 1), using NADH as cofactor.	DHPR deficiency results in progressive mental retardation, neuronal loss, abnormal vascular proliferation in the brain, basal ganglia calcification and sudden death [110]. It accounts for 33% of BH_4_ deficiencies, with ∼303 cases reported so far [110].
DnaJ homolog subfamily C, member 12 (DNAJC12); *DNAJC12*	DNAJC12 is an Hsp40 co-chaperone with a critical role in maintaining the protein homeostasis of the AAAHs, including PAH and TH [14].	DNAJC12 deficiency results in a multitude of neurological symptoms, including developmental delay, intellectual disability, infantile dystonia and parkinsonism [14,112,113]. So far, ∼52 patients have been described [114].
Aromatic L-amino acid decarboxylase (AADC or AAAD); *DDC*	AADC decarboxylates L-DOPA to form DA (Figure 1).	AADC deficiency is clinically characterized by infantile hypotonia, ophthalmic crisis and developmental delay [115].
Dopamine transporter (DAT); *SLC6A3*	DAT regulates DA homeostasis by transporting extracellular DA into the intracellular space (Figure 1). DAT is a key regulator of the amplitude and duration of dopaminergic transmission [116].	DAT deficiency leads to neuropsychiatric disorders and neurodegeneration [117], with a phenotype characterized by an infantile-onset hyperkinetic movement disorder [118,119]. Ng et al. reported 31 patients [119].
Vesicular monoamine transporter type 2 (VMAT2); *SLC18A2*	VMAT2 transports DA from the cytosol into synaptic vesicles, for their subsequent release from the neuron during neurotransmission (Figure 1). Moreover, the sequestration of cytosolic DA into vesicles prevents neuronal damage [120].	Clinical symptoms of VMAT2 deficiency include infantile-onset movement disorder, mood disturbance, autonomic instability, and developmental delay [121]. Twelve patients were reported by Padmakumar et al. [122].

## Current treatment options

While the increasing understanding of the pathogenic mechanisms behind parkinsonism has unveiled crucial pathways that can be targeted for the development of potential therapies, current treatments primarily aim at managing symptoms, but do not directly address the underlying molecular mechanisms that drive disease progression. Existing treatments, like levodopa, transiently elevate DA levels in the brain (Figure 1), since the conversion of the DA precursor L-DOPA to DA is not normally rate-limiting [29,30]. While levodopa is effective for the management of motor symptoms, its pharmacokinetics presents high interindividual variability and unpredictable plasma levels, leading to increased administration and unavoidable side effects, such as dyskinesia, after several years of treatment [31]. Thus, modifying the pharmacokinetics of levodopa by optimizing its delivery for prolonged release is a pursued strategy to alleviate the deterioration associated with prolonged treatments. This can be achieved by using innovative oral or non-oral delivery systems for extended-release, or by combining levodopa with other therapies [32]. So far, the most common approach is the co-administration with inhibitors of either AADC (i.e. carbidopa or benserazide) and/or peripheral COMT (i.e. tolcapone and entacapone). This prevents the peripheral degradation of levodopa before it reaches the brain, increasing its brain bioavailability and enhancing the effectiveness of the treatment [33]. Other approved approaches aiming to stabilize DA in the neuron, either independently or as an adjunct therapy to levodopa, are the use of MAO-B inhibitors (i.e. rasagiline and selegiline) [33]. Finally, DA receptor agonists like pramipexole and ropinirole bind with high-affinity to the D2 and D3 DA receptors, mimicking the actions of DA in the brain and effectively increases DA signaling [34] (Figure 1).

## Novel therapies

**Figure 2.**
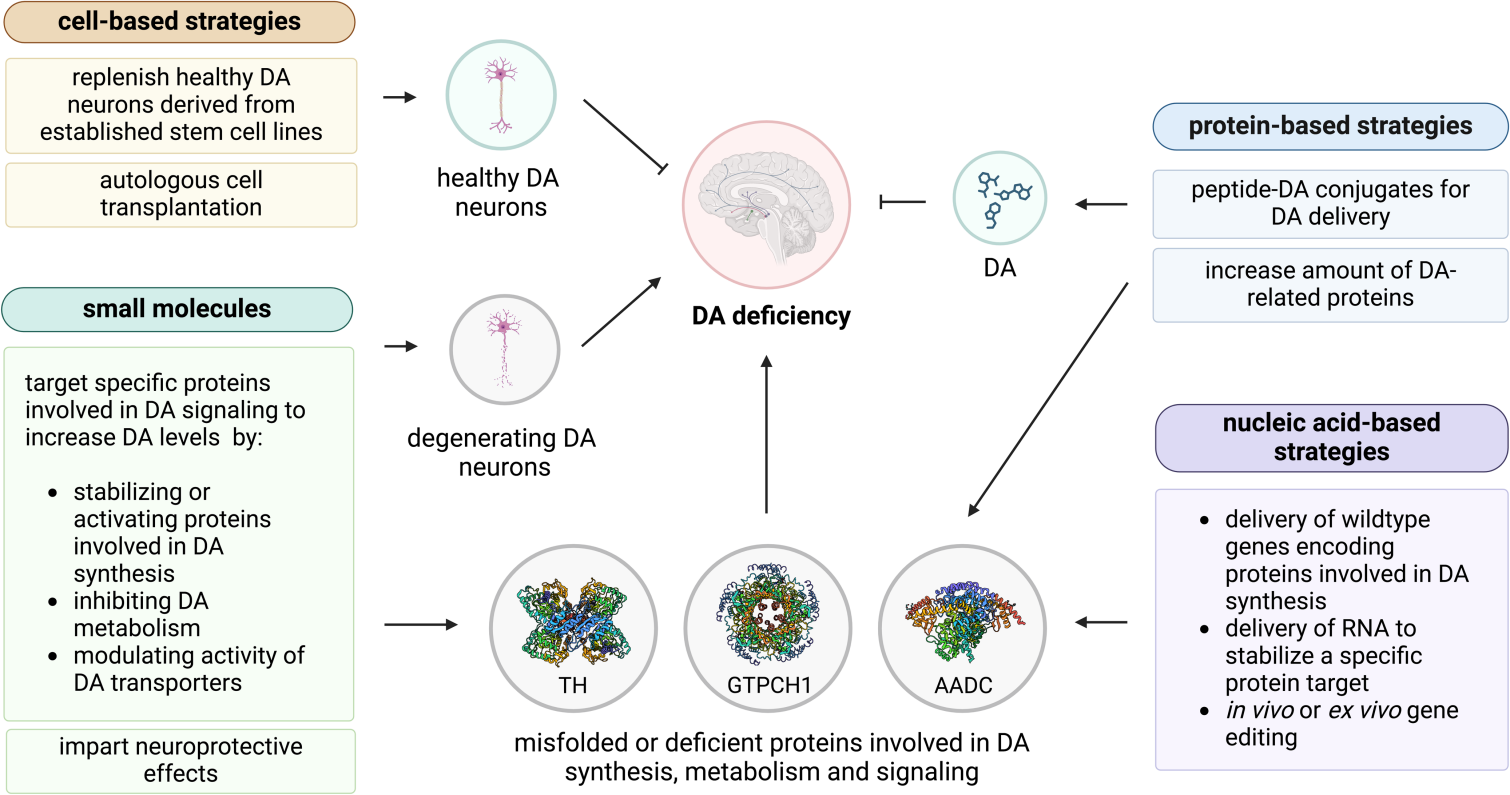
Overview of novel therapies that aim to increase DA synthesis and signaling, focusing on cell-, nucleic acid-, and protein-based strategies, and small molecules. Cell-based strategies aim to increase DA by replenishing DA producing neurons in patients with neurodegeneration. These cells can either be derived from established cell lines or from the patients themselves. Nucleic acid-based strategies on the other hand, aim to increase wildtype proteins by delivering genes to compensate for deficient proteins, by using RNA to stabilize protein targets or by editing variants in parkinsonisms with genetic etiology. Protein-based strategies aim to increase the levels of deficient proteins, and peptides can also act as carriers to deliver DA. Lastly, small molecule compounds can impart general neuroprotective effects, or stabilize or modulate the activity of specific proteins involved in DA signaling.

Numerous therapies are currently being developed that not only aim to alleviate symptoms but also target the correction of DA signaling (Figure 2). Decreased DA signaling is mainly caused by insufficient amounts of DA, inefficient signal transmission or death of dopaminergic neurons. In this chapter, we provide a brief overview of innovative therapeutic strategies that target these dysregulated processes.

### Cell-based therapies

PD is the most common cause of parkinsonism, and the second most common progressive neurological disorder after Alzheimer's disease. PD is characterized by the loss of dopaminergic neurons in the *substantia nigra* and the consequential striatal DA deficiency [35]. It appears that 50–80% of the dopaminergic neurons are already lost by the time motor symptoms become apparent in PD patients [35,36]. Although the pathogenic mechanisms in PD are not clear, mitochondrial dysfunction, oxidative stress, neuroinflammation and loss of protein homeostasis are affected in idiopathic PD and appear involved in the dopaminergic neuronal death process [3]. Thus, in parallel with efforts to develop therapies that specifically target these pathways to prevent and delay PD progression [37,38], cell-based strategies are also being developed to increase DA signaling in patients that already have substantial loss of DA-producing neurons [39].

The transplantation of fetal mesencephalic tissue, a region rich in dopaminergic neuroblasts, to the DA-depleted striatum in patients with PD is one strategy to restore DA neurotransmission. While this approach increased DA signaling and ameliorated motor symptoms in patients with advanced PD, these improvements were not clear with respect to positive clinical outcome [40,41], but a sustained improvement in motor control and movement was however observed even 15–18 years post-operatively [42]. Nevertheless, ethical concerns regarding transplantation of fetal mesencephalic tissue from donors and issues in acquiring a reliable supply of cells have hampered the development and wide use of this treatment, thus alternative cell sources, such as stem cells, are also being explored.

Stem cells are self-renewing and can differentiate into a variety of cell types. In particular, the use of pluripotent stem cells (PSCs) poses less ethical concerns and risks as the cells are easier to acquire and are amenable for autologous transplantation in some situations. These PSCs can either be derived from developing blastocysts (embryonic stem cells; ESCs) or reprogrammed from somatic cells (induced PSCs; iPSCs). Transplantation of dopaminergic neuron progenitor cells derived from an established human ESC line to the putamen of mice has been shown to be efficacious [43], safe, well-tolerated and effective with respect to clinical outcome in the Phase 1 clinical trial 1 year post-operatively [44]. A study conducted in the monkey-model of PD showed improved motor and non-motor symptoms with extensive neuron axon growth and strong DA activity 2 years after autologous transplantation of rhesus iPSC-derived dopaminergic progenitor cells, which does not require immunosuppression [45]. The autologous co-transplantation of regulatory T-cells with iPSC-derived dopaminergic neurons has also been recently found to increase transplanted cell survival in mice, presenting a promising strategy to further improve clinical outcomes of cell-based therapies for PD [46].

### Nucleic acid-based therapies

Nucleic acid-based therapeutic strategies aim to treat diseases by modulating gene expression or restoring the normal function of gene products through gene editing, or by delivering DNA or mRNA through viral or non-viral approaches. One strategy to enhance DA signaling and treat parkinsonism is gene therapy, where DNA encoding for enzymes that may restore DA synthesis is delivered to the brain, using both adeno-associated virus and lentivirus to deliver the genes. The initial gene therapy trials for PD focused on the introduction of the *AADC* gene into specific target neurons to enhance the conversion of administered L-DOPA to DA [47]. More recently, preclinical and clinical studies have investigated the enhancement of DA production and signaling by transducing the *TH*, *AADC*, *GCH1* genes together into non-degenerating neurons. Although no therapy has yet been approved, on-going clinical trials support the potential of the approach [48,49]. The efficacy of gene therapy using viral vectors is also being proven in inherited disorders associated with DA deficiency and parkinsonism. Ongoing preclinical and clinical gene therapy studies for inherited disorders of neurotransmitter metabolism using viral vectors include successful preclinical studies targeting AADC-, DAT- and PTPS-deficiencies, as well as interventional clinical trials for AADC [50].

With respect to RNA-therapies, full-length PAH mRNA encapsulated in nanoparticles seems effective in restoring PAH levels and L-Phe metabolism in PAH-deficient mice, demonstrating the possibility of increasing protein expression [51]. Moreover, the long non-coding RNA *HULC* has been reported to modulate its activity by facilitating PAH-substrate and PAH-cofactor interactions, thus potentiating the enzyme for L-Phe metabolism [52]. Shorter *HULC* mimics also showed corrective effect of L-Phe levels in mice carrying a *PAH* disease variant [52].

CRISPR-Cas9-based gene editing is also emerging as an alternative approach to correct pathogenic variants *in vivo*. Moreover, the safety and therapeutic potential of combining gene editing *ex vivo* and stem cell therapy has been demonstrated by exagamglogene autotemcel (exa-cel or Casgevy), the first *ex vivo* gene therapy for sickle cell anemia and the first CRISPR-Cas9-based therapy approved in the US [53]. In the case of PD, as well as monogenic forms of parkinsonism, CRISPR-based genome editing also appears promising and research is underway to correct the predominant genetic variant in glucocerebrosidase (*GBA*), linked to an elevated risk of developing PD, in both Gaucher's patients and asymptomatic carriers [54]. The effectiveness of this approach was also investigated preclinically in a mouse model with classic PKU, where rescue of the disease phenotype was observed after a single base change in *PAH* [55].

### Protein-based and enzyme replacement therapies

Protein-based therapeutic strategies traditionally involve the targeted delivery of proteins or peptides to replace or modulate the activity of their target. A potential approach to treat parkinsonism is the administration of functional proteins to compensate for their dysfunction (also known as enzyme replacement therapy or ERT). Functional full-length TH has been successfully delivered to dopaminergic cells in culture and to mouse brain using maltodextrin nanoparticles, leading to increased L-DOPA synthesis [56]. Interestingly, an artificial TH-mimicking Fe_3_O_4_ nanozyme that hydroxylates L-Tyr to L-DOPA and that also includes transferrin receptor aptamers to allow efficient crossing of the BBB and internalization into neurons has been prepared [57]. The device also includes a block strand with antisense oligonucleotides to α-synuclein (*SNCA*) mRNA to allow the specific targeting of degenerating dopaminergic neurons, as well as antisense oligonucleotide treatment and fluorescence imaging. Treatment of a PD mouse model with FNA-Fe_3_O_4_ resulted in recovery of L-DOPA and DA levels and amelioration of motor symptoms [57].

### Small molecules

Small molecules still dominate the pharmaceutical industry at present despite the development of biologics (e.g. cell-, gene- or protein- based therapies) due to easier administration, amenability to be produced through chemical and bioengineering processes, cheaper costs, stability and ability to evade the immune response. There are numerous drug discovery initiatives underway, from screening of extensive compound libraries against predefined targets to the investigation of potent natural products for the identification of novel active agents. Below, we discuss ongoing efforts to identify small molecules to treat parkinsonism that focus on increasing DA levels and DA signaling in the brain. A selection of these compounds is presented in Table 2.

**Table 2.** Names, chemical structures, targets and detailed activities of small molecules aimed at increasing DA levels and DA signaling in the brain, offering potential as treatments for parkinsonism

Small molecules activating/stabilizing enzymes involved in DA signaling			
Name	Structure	Target	Activity
Compound III (3-amino-2-benzyl-7-nitro-4-(2-quinolyl)-1,2-dihydroisoquinolin-1-one)	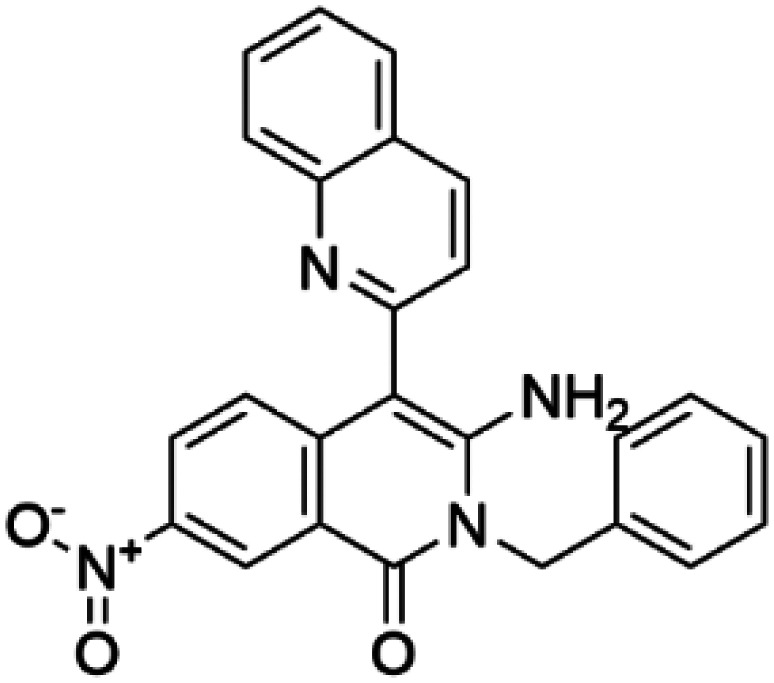	PAH	Compound III stabilizes PAH *in vitro* and increases activity and steady-state protein levels in cells and mice [63].
		TH	Compound III stabilizes TH *in vitro* and increases total TH activity in mouse brains [68].
Compound IV (5,6-dimethyl-3-(4-methyl-2-pyridinyl)-2-thioxo-2,3-dihydrothieno[2,3-d] pyrimidin-4(1H)-one)	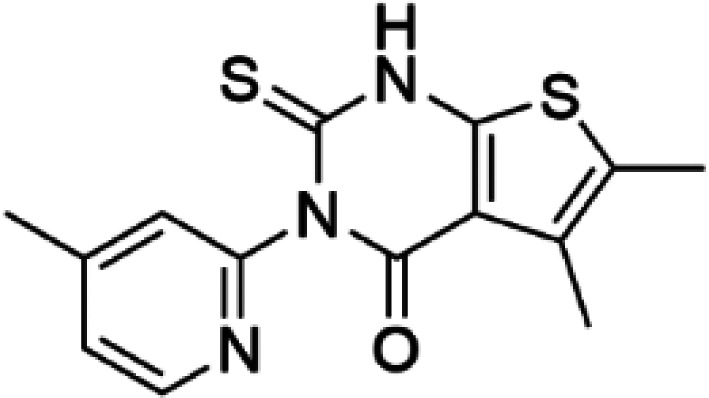	PAH	Compound IV stabilizes PAH *in vitro* and increases activity and steady-state protein levels in cells and mice [63,69].
Compound 2 ((2E)-3-(2-chlorophenyl)-N-(4-[(3,4-dimethyl-1,2-oxazol-5-yl) sulfamoyl]phenyl) acrylamide)	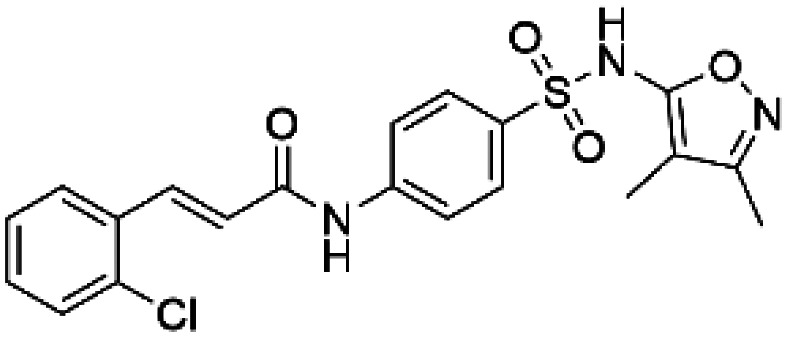	TH	Compound 2 stabilizes TH *in vitro* and increases the activity and steady-state protein levels in cells [64].
Compound 4 (N-allyl-2-(4-oxo-3,4,5,6,7,8-hexahydro[1] benzothieno[2,3-d]pyrimidin-2-yl) hydrazinecarbothio amide)	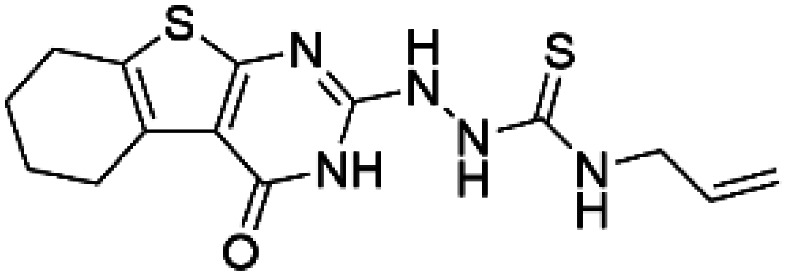	TH	Compound 4 increases the half-life of the TH activity *in vitro* [64].
Compound 5 ((7S)-2,7-dimethyl-5,6,7,8-tetrahydro[1] benzothieno[2,3-d]pyrimidin-4(3H)-one)	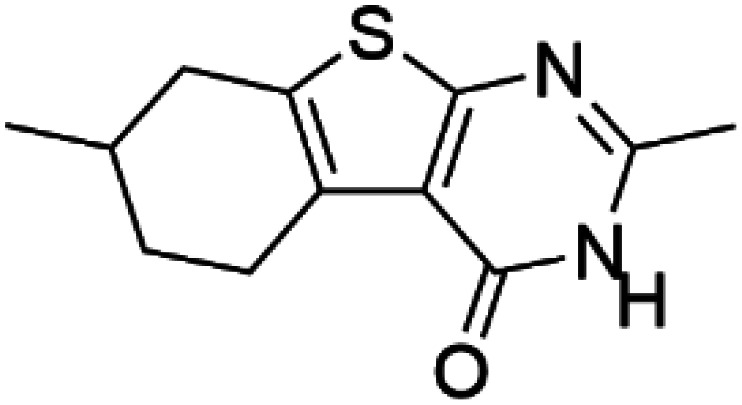	TH	Compound 5 stabilizes TH *in vitro* and increases activity and steady-state protein levels in cells [64].
Levalbuterol	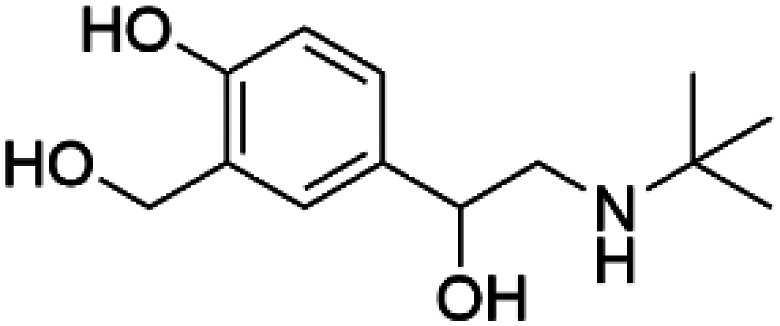	TH	Levalbuterol is an FDA-approved drug that stabilizes TH, lowers the feedback inhibition by DA and delays misfolding and aggregation of TH *in vitro* [65].
Ibogaine	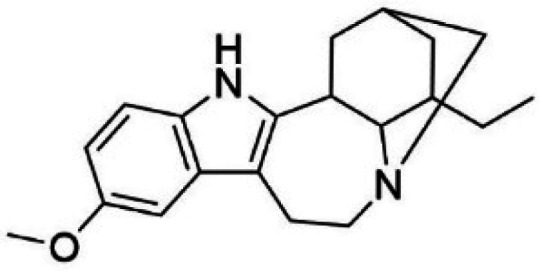	DAT	Ibogaine restores the folding of DAT, increases its surface expression and enhances DA transport in cellular and *in vivo* models [70,71].
Noribogaine	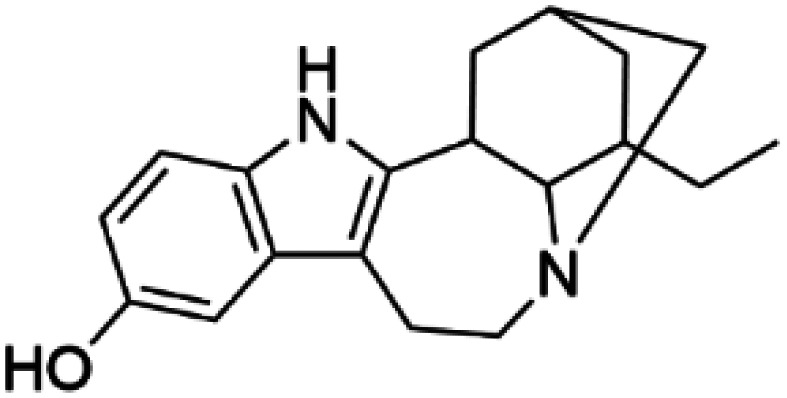	DAT	Noribogaine restores the folding of DAT and enhances DA transport in cellular and *in vivo* models [70].
Bupropion	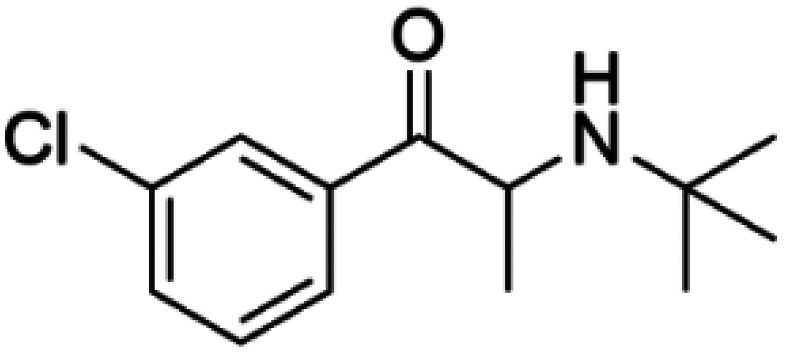	DAT	Bupropion is an FDA-approved drug that increases the surface expression of DAT and enhances DA uptake activity in cells [71].
Sapropterin dihydrochloride (orally active synthetic form of BH_4_)	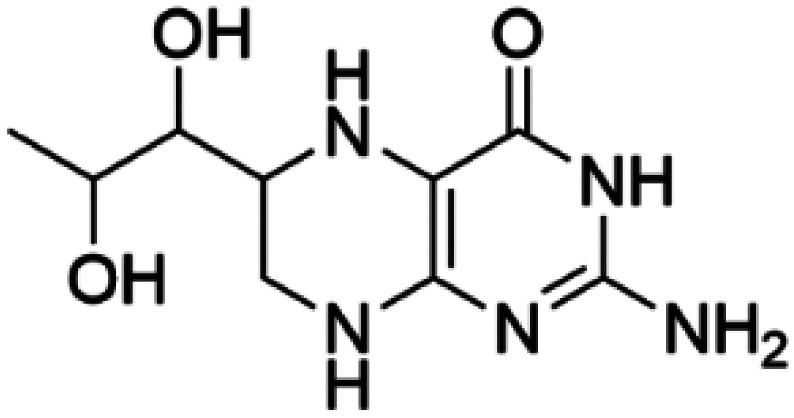	GTPCH1, PTPS, DHPR, PCD, SPR, PAH, TH	BH_4_ supplementation therapy (sapropterin dihydrochloride, Kuvan®) is used for BH_4_ deficiencies [79] and PAH deficiency [80]. For TH deficiencies, BH_4_ has demonstrated to stabilize TH *in vitro* and *in vivo*, increasing TH levels and activity [81] and improves phenotype in human iPSCs and in the THD mouse model [82].

Small molecules with neuroprotective activity		
**Name**	**Structure**	**Activity**
Epoxide 4	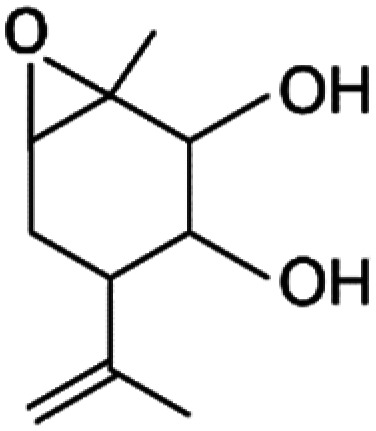	Epoxide 4 promotes the survival of cultured dopaminergic neurons and protect them against degeneration. In the PD mouse model, Epoxide 4 increases the density of DA neuron fibers in the striatum [83].
MCC950 (N-((1,2,3,5,6,7-hexahydro-s-indacen-4-yl) carbamoyl)-4-(2-hydroxypropan-2-yl) furan-2-sulfonamide)	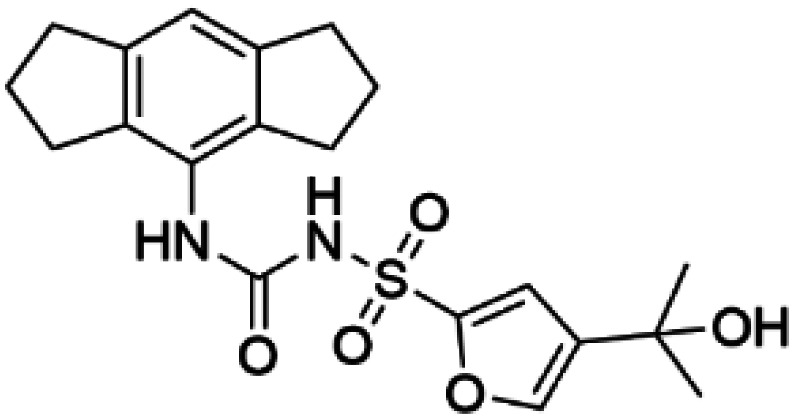	MCC950 mitigates motor deficits and nigrostriatal dopaminergic degeneration in multiple rodent PD models [84].
BRF110 (4-[Methyl[2-phenyl-5-(2-propen-1-yl)-6-(trifluoromethyl)-4-pyrimidinyl]amino] benzoic acid)	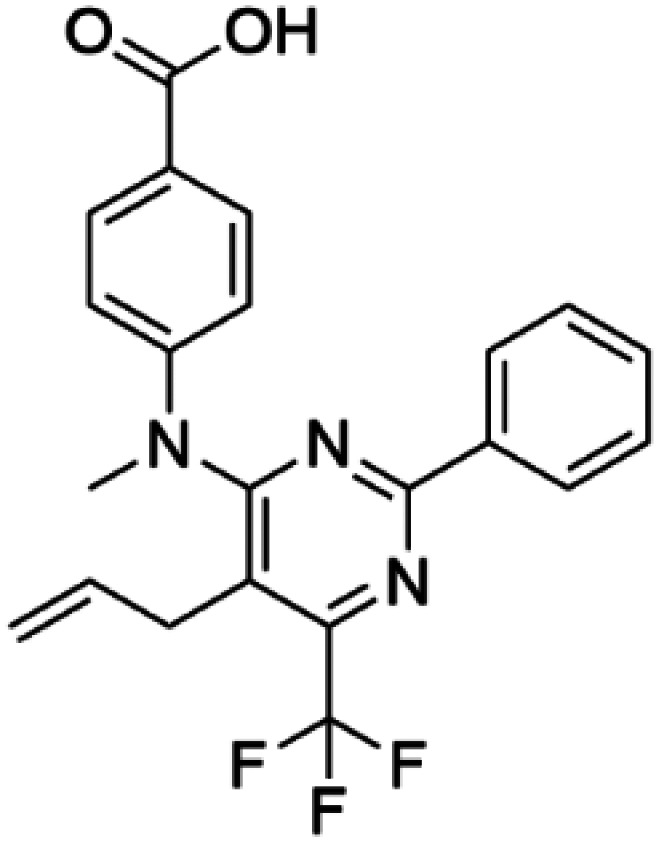	BRF110 prevents dopaminergic neuron demise and striatal dopaminergic denervation *in vivo*. Remarkably, besides neuroprotection, BRF110 up-regulates *TH*, *DDC* and *GCH1* transcription and increases striatal DA in the PD mouse model [85].
IRX4204 ((2E,4E)-3-Methyl-5-[(1S,2S)-2-methyl-2-(5,5,8,8-tetramethyl-5,6,7,8-tetrahydro-2-naphthalenyl) cyclopropyl]-2,4-pentadienoic acid)	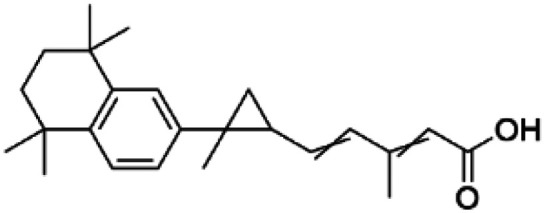	IRX4204 promotes the survival and maintenance of nigral DA neurons in primary mesencephalic cultures, and attenuates motor deficits in a rat model of PD [86].
Fasudil	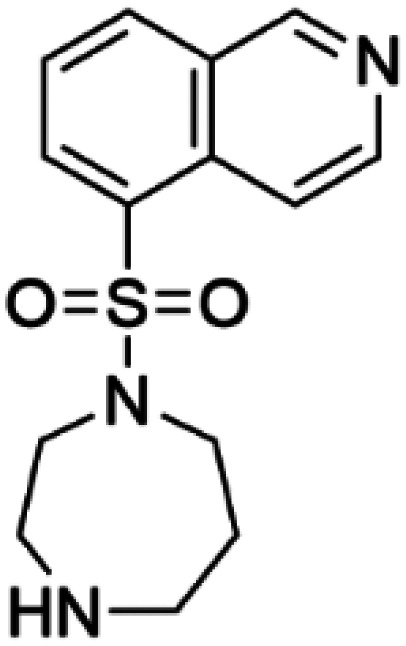	Fasudil, an FDA-approved drug, reduces dopaminergic cell loss and improves motor function in a mouse model of PD [87].
Lapatinib	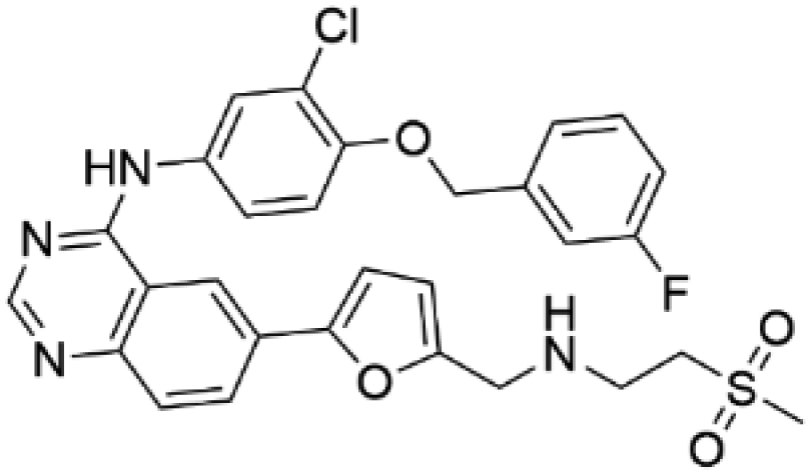	Administration of Lapatinib in a rat model of PD improves motor function and restores dopaminergic neurons by increasing TH expression, leading to elevated DA levels [88].
Pedicularioside A	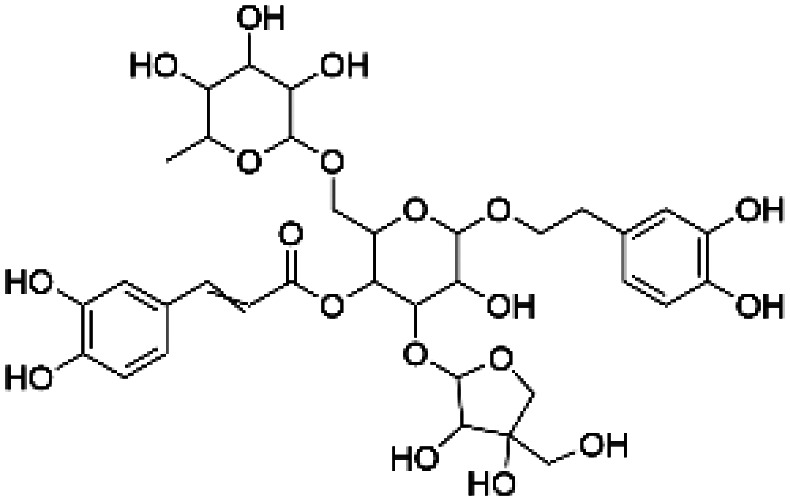	Pedicularioside, a plant-derived terpene, has been shown to prevent the loss of DA neurons in a PD neuron model [90].
Celastrol	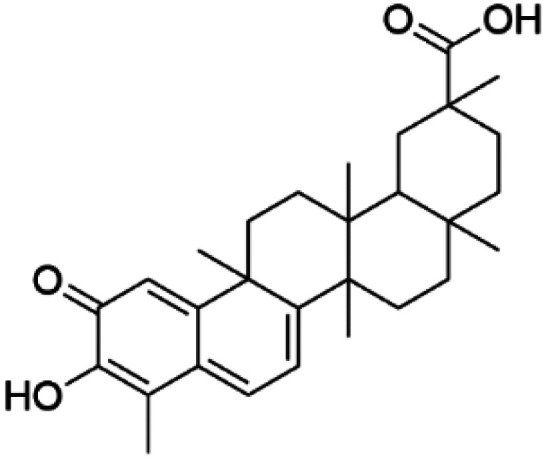	Celastrol, a plant-derived terpene, provides neuroprotection in PD cell models and also improves motor symptoms in PD mouse model [91].
Curcumin	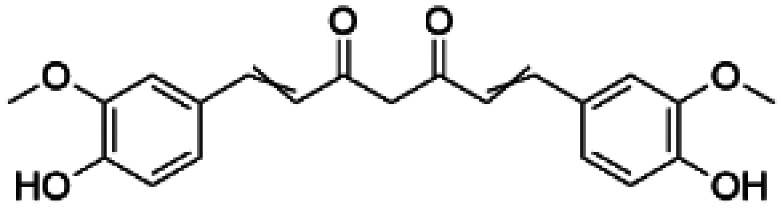	Curcumin is a potent antioxidant from turmeric plant, with protects dopaminergic neurons and improves motor behavioral performance in PD animal models [92,93].
Epigallocatechin-3-gallate	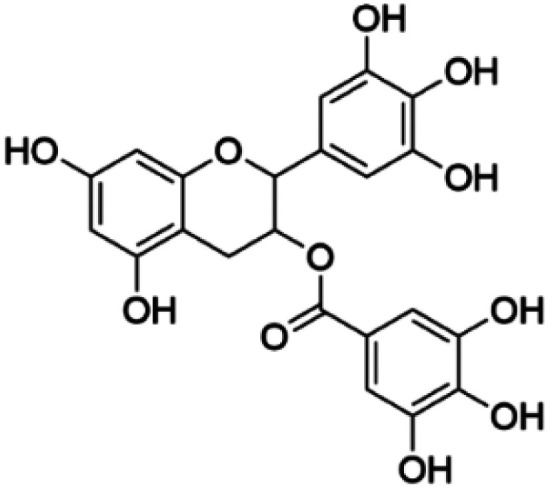	A polyphenolic component extracted from green tea, which has shown neuroprotective effects in cellular and animal models of PD, through different molecular mechanisms such as the modulation of DA production [94].
Resveratrol	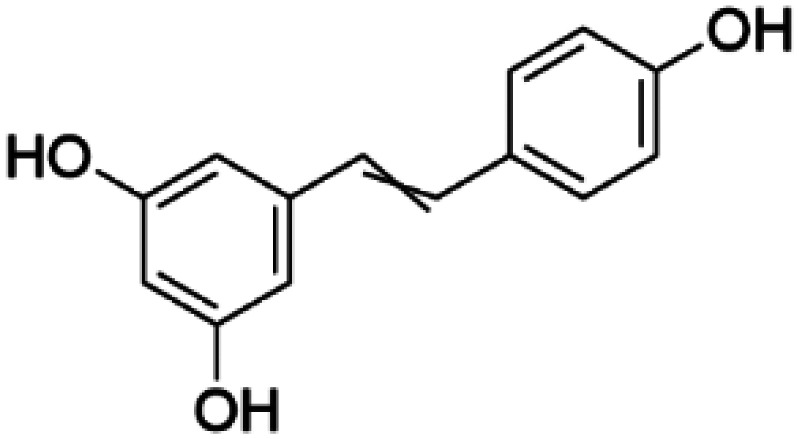	Resveratrol, a polyphenol contained in blueberries or grapes, exhibits potent neuroprotective activity in cellular and animal models of PD [95].

**Small molecules inhibiting VMAT2**			
**Name**	**Structure**	**Target**	**Activity**
Tetrabenazine	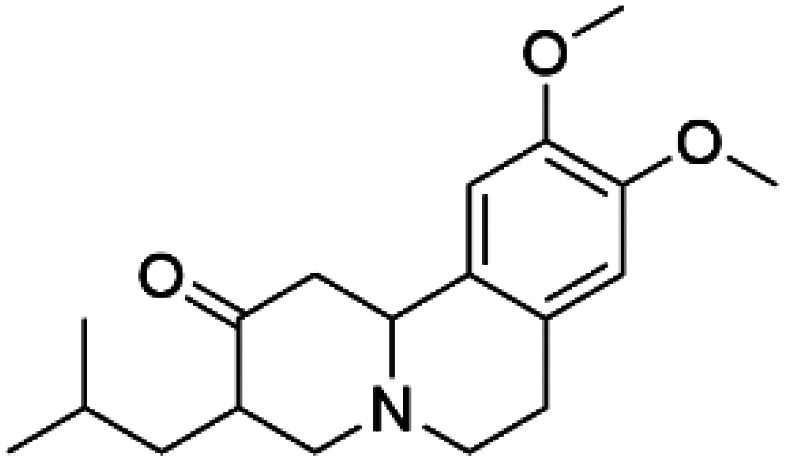	VMAT2	Tetrabenazine is an approved inhibitor of VMAT2 for the treatment of chorea associated to Huntington's disease [99]. Salmeterol and ziprasidone are FDA-approved compounds able to inhibit VMAT2, which could potentially be repurposed for the symptomatic treatment of tardive dyskinesia and other hyperkinetic movements [102].
Salmeterol	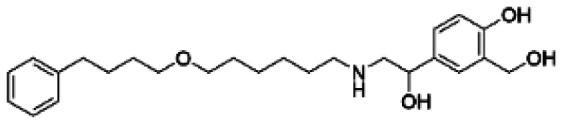		
Ziprasidone	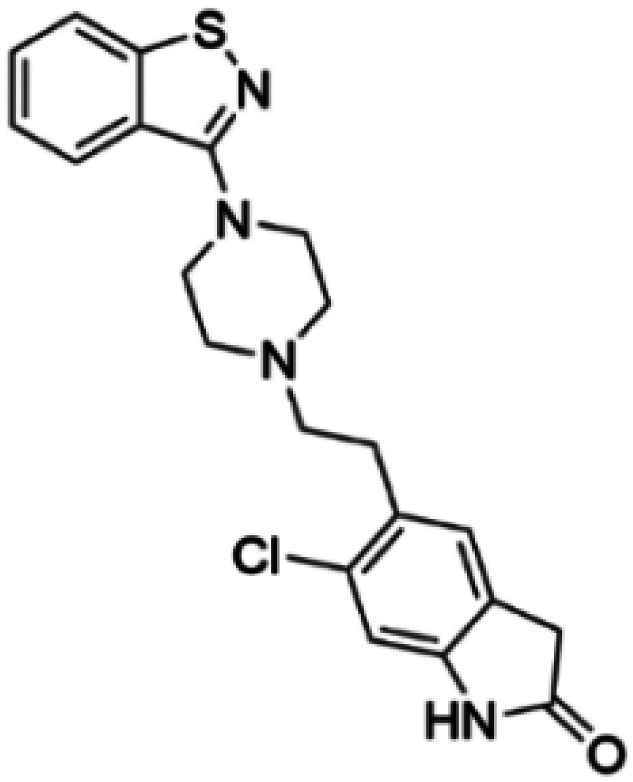		

The small molecules are classified into three groups according to their functions: activators and stabilizers of enzymes involved in DA synthesis and signaling, small molecules with neuroprotective activity and inhibitors of VMAT2.

Small molecules are low-molecular mass organic compounds that selectively bind to their target proteins, resulting in modulation of conformation and/or activity. The interest in the development of compounds that restore function by correcting the folding and/or increasing stability to protect the targets from accelerated degradation (pharmacological chaperones, PCs) for the treatment of inborn error of metabolism and other misfolding diseases is increasing [58–60]. Successful therapies for Fabry's disease (Migalastat), transthyretin amyloidosis (Tafamidis), cystic fibrosis (Elexacafor and Tezakafor) have strengthened the PC-field. One of the most common strategies for identifying PCs is by high-throughput screening (HTS), which allows rapid testing of large compound libraries against a pre-selected target. HTS can be performed with biophysical screens, using purified proteins as targets, or cellular screens either monitoring the cellular phenotype or changes in protein levels [61]. This methodology is normally applied using large collections of diverse drug-like compounds or FDA-approved drugs, in this latter case leading to the possibility of drug repurposing, which may accelerate the drug development timeline and reduce costs related to hit development and safety testing [62].

HTS using differential scanning fluorimetry (DSF) to monitor the binding of compound hits to specific targets has been effective to identify compounds with PC capability for DA deficiencies, especially for PAH and TH [63–65]. Other screening methods relying on affinity-immobilization, immunoprecipitation and measurement of PAH activity by tandem mass spectrometry have also been developed [66]. In the case of PAH, HTS of 1000 compounds randomly selected from a larger diversity library identified two PCs (compounds III and IV) that stabilized PAH *in vitro*, increased activity and steady-state protein levels of wildtype and variant PAH in cells and mice [63]. Later, a new generation of compound IV-analogues producing higher affinity and stabilization of PAH were developed by alchemical design [67]. Compounds III and IV (Table 2) were also investigated for their potential activity on the other BH_4_ dependent AAAHs, TH and TPH2 [68], as these hydroxylases are highly-conserved in sequence, structure and catalytic mechanism. Compound III stabilized TH *in vitro* and increased total TH activity in mouse brains [68], representing a valuable proof of concept for the potential of PCs as therapies for the treatment of THD and other parkinsonisms. However, as this compound is not specific for TH, additional HTS runs were performed to identify more effective and specific compounds. HTS-DSF of 10 000 compounds identified three additional hits (compounds 2, 4 and 5; Table 2) that stabilized TH *in vitro*, with compound 5 additionally increasing TH activity when studied in cells transiently transfected with THD variants [64]. EPR, X-ray crystallization and docking revealed that compounds 4, 5 and previous compound IV constitute a group of binders that weakly coordinate via a pyrimidine nitrogen to the catalytic iron of TH without significant inhibition, and seem to protect the iron from rapid inactivation and formation of reactive oxygen species [64,69]. Levalbuterol (Table 2), a TH stabilizer obtained from HTS-DSF of the Prestwick chemical library of FDA-approved drugs, also seems to exert a similar protective mechanism by coordinating to the iron [65]. Despite their stabilizing effect on TH *in vitro*, the low affinity of these PCs implies too high doses to obtain *in vivo* proof of concept, and the search and design of more effective PCs, binding outside the active site to stabilize TH and variants specifically and effectively, continues. Another dopaminergic target for which PCs has been investigated is DAT. The impact of ibogaine and noribogaine (Table 2), PCs for the closely related serotonin transporter, was studied for the folding of DAT. These compounds demonstrated the ability to bind to the inward-facing DAT conformation, effectively restoring its folding and thereby enhancing DA transport in disease-related variants in both cellular and *in vivo* models [70]. Ibogaine and bupropion were also identified as compounds that increased the surface expression of DAT, enhanced DA uptake activity, and promoted the maturation of DAT protein in some disease-mutants in cells [71]. More recently, a series of analogues of ibogaine have been generated to find more effective PCs for DAT deficiencies [72,73]. A novel strategy to address DA deficiencies includes not only focusing on the enzymes responsible for DA synthesis or metabolism, but also addressing the molecular partners crucial for their homeostasis. For example, TH interacts with regulatory proteins such as 14-3-3 [74,75], GTPCH1 [76] and DNAJC12 [14] (Figure 1), which play essential roles in maintaining TH stability. The search for compounds that can modulate these protein–protein interactions is also emerging as a promising avenue for the development of alternative therapeutic approaches [25,77]. Likewise, enzyme cofactors are also considered to have PC potential for their target enzymes [78]. BH_4_ supplementation therapy (sapropterin dihydrochloride, Kuvan®) (Table 2) is used not only for BH_4_ deficiencies [79], but also is one of the option treatments for BH_4_ responsive PAH deficiency [80]. For TH deficiencies, BH_4_ has been found to stabilize TH, wildtype and variants, *in vitro* and *in vivo*, increasing TH levels and activity [81]. Recently it has also been shown that BH_4_ treatment improves THD phenotype in human iPSCs and in the THD mouse model [82].

Other small molecules have shown potential to develop and maintain dopaminergic neurons, such as Epoxide 4 [83], MCC950 [84], BRF110 [85] and IRX4204 [86] (Table 2). These molecules have been effective in increasing the survival of DA neurons, raising DA content in the brain, and alleviating motor symptoms in experimental PD animals. Furthermore, two FDA drugs approved for other applications, i.e. Fasudil [87] and Lapatinib (Table 2) [88], have been shown to boost striatal DA levels, enhance dopaminergic neuronal survival and improve the motor function in PD animal models. Fasudil is being evaluated in a clinical trial in PD patients (EudraCT: 2021-003879-34).

A large fraction of small molecules is extracted from natural compounds. Some natural products display well-known anti-oxidative and anti-inflammatory capabilities, inhibit protein misfolding, and boost mitochondrial homeostasis and other neuroprotective processes [89]. Notably, terpenes such as pedicularioside A [90] and celastrol [91] (Table 2), have shown efficacy in preventing the loss of dopaminergic neurons in both *in vitro* and *in vivo* studies. Additionally, polyphenols such as curcumin [92,93], epigallocatechin-3-gallate [94] or resveratrol [95] (Table 2) have also been demonstrated to provide neuroprotection through different pathways, exhibiting positive effects on motor and behavioral function by promoting the survival of dopaminergic neurons and increasing DA levels in different PD models [96,97].

Finally, it is worth mentioning that the general pathomechanisms in parkinsonisms rather infer the need of therapies that increase function of dysregulated or dysfunctional targets (both in PD and in the inherited disorders in Table 1), and most of the therapies discussed above thus refer to activating/stabilizing approaches. However, there are some instances with need of novel inhibitors. For example, in the treatment of tardive dyskinesia (TD) and other hyperkinetic movements, it may be beneficial to inhibit VMAT2 activity to reduce DA storage and release from pre-synaptic neurons into the synaptic cleft, reducing stimulation of post-synaptic DA receptors and subsequently decreasing dyskinetic movements. Reserpine is a known VMAT2 inhibitor that is used as an antipsychotic and for the symptomatic treatment of chorea associated to Huntington's disease [98], but it is no longer used today due to adverse side effects. Tetrabenazine [99] (Table 2), and its variants deutetrabenazine and valbenazine are also VMAT2 inhibitors that have been approved by the FDA for treating TD [100,101], but efforts continue to develop better VMAT2 inhibitors. Interestingly, target based HTS using DSF to monitor ligand binding can also be successfully applied to the discovery of inhibitors, after the hit binders are further evaluated in specific functional assays. This strategy was used for the identification of several approved drugs as novel VMAT2 inhibitors, such as salmeterol or ziprasidone (Table 2), with great potential to be repurposed for the symptomatic treatment of TD in PD and other conditions [102].

## Conclusions

DA is a neurotransmitter that plays an essential role in many important body functions such as movement, memory, and pleasure. Insufficient levels of DA can lead to a spectrum of mental and physical health issues and contribute to the development of parkinsonisms. The current treatment to address DA deficiencies involves supplementation with levodopa, which is converted to DA in the brain. While levodopa effectively manages symptoms, it does not offer a cure for the underlying condition. Consequently, it is essential to investigate alternative treatment strategies to target the fundamental pathomechanisms of DA deficiencies. Numerous molecular mechanisms can disrupt DA levels in the brain, therefore several strategies and targets should be considered. Advances in understanding the molecular basis of different DA deficiencies have identified new therapeutic targets and promising disease-modifying strategies. However, translating these research findings into tangible improvements in patient care remains a significant challenge. Many of the promising strategies have yet to reach human clinical trials, primarily due to challenges related to pharmacokinetics, pharmacodynamics, safety and toxicity. Future research should address these aspects to facilitate the translation of scientific discoveries into practical and effective treatments for patients.

## Perspectives

- DA is an essential neurotransmitter involved in behavioral, cognitive, and motor functions. Deficiencies in DA lead to debilitating motor disorders, such as dystonia and parkinsonism. Pediatric inherited forms of these disorders often include neurodevelopmental and cognitive impairments.
- The currently available treatments for DA deficiencies are primarily based on levodopa and exhibit variable efficacy, only addressing symptoms. Alternative treatment strategies that target the fundamental pathomechanisms of DA deficiencies are highly needed.
- Novel disease-modifying therapeutic approaches, rooted in advancing insights into the underlying pathogenic mechanisms affecting DA synthesis and transport, hold great promise. These approaches aim not only to address parkinsonism but also to mitigate neurodevelopmental and cognitive impairments.

## Abbreviations

AAAH: aromatic amino acid hydroxylase
AADC: aromatic acid decarboxylase
AD: autosomal dominant
DA: dopamine
DAT: dopamine transporter
DHPR: dihydropteridine reductase
DSF: differential scanning fluorimetry
ESC: embryonic stem cell
HTS: high-throughput screening
iPSC: induced PSC
MAO: monoamine oxidase
PAH: phenylalanine hydroxylase
PC: pharmacological chaperone
PD: Parkinson's disease
PSC: pluripotent stem cell
TD: tardive dyskinesia
TH: tyrosine hydroxylase
